# Misophonia and Potential Underlying Mechanisms: A Perspective

**DOI:** 10.3389/fpsyg.2018.00953

**Published:** 2018-06-29

**Authors:** Devon B. Palumbo, Ola Alsalman, Dirk De Ridder, Jae-Jin Song, Sven Vanneste

**Affiliations:** ^1^Lab for Clinical and Integrative Neuroscience, School of Behavioral and Brain Sciences, The University of Texas at Dallas, Richardson, TX, United States; ^2^Department of Surgical Sciences, Section of Neurosurgery, Dunedin School of Medicine, University of Otago, Dunedin, New Zealand; ^3^Department of Otorhinolaryngology-Head and Neck Surgery, Seoul National University Bundang Hospital, Seongnam, South Korea

**Keywords:** misophonia, auditory system, limbic system, associative learning, classical conditioning, sensitization

## Abstract

There is a growing research interest in the diagnosis rate of misophonia, a condition characterized by a negative emotional/autonomic reaction to specific everyday sounds. Diagnosis of misophonia requires a thorough case history and audiological test procedures. Associative and non-associative learning models for understanding the underlying mechanisms of misophonia have been presented. Currently, there is no cure or pharmaceutical agent for misophonia; however, therapy programs addressing misophonia and its characteristics do exist. Investigation of comorbid conditions and other psychological therapy strategies might help to reveal more about the underlying mechanisms and potentially lead to a successful treatment method.

## Introduction

Misophonia is a condition where patients experience a negative emotional reaction and dislike (e.g., anxiety, agitation, and annoyance) to specific sounds (e.g., ballpoint pen clicking (repeatedly), tapping, typing, chewing, breathing, swallowing, tapping foot, etc.) (Jastreboff and Jastreboff, 2002). Misophonia is a derivate from the Greek words misos (hate) and phónè (voice), and means hate of sound. Each patient’s reaction is unique as it depends on the specific conditions under which the sound was experienced and any previous evaluations of that sound. Prior to Jastreboff introducing the term misophonia, there have been different terms to describe the condition, such as soft sound sensitivity symptom, select sound sensitivity syndrome, decreased sound tolerance, and sound-rage (Schwartz et al., 2011; Neal and Cavanna, 2013). A patient’s negative reaction and dislike may occur in response to sound at any level. Although hyperacusis and misophonia can coexist, hyperacusis refers specifically to an increased sensitivity to certain frequencies and volume ranges of sound (Song et al., 2014). Misophonia can be distinguished from hyperacusis by its sensitivity to the subjective response provoked (Pienkowski et al., 2014). A subtype of misophonia is phonophobia, when fear to a specific sound is the dominant factor (Jastreboff and Hazell, 1999; Henry et al., 2002; Jastreboff and Jastreboff, 2015). It is important to recognize that “subtype” implies that the class of sounds that elicit phonophobia are drawn from misophonic sounds or that they share a similar mechanism, neither of which is necessarily true. From a phenomenological viewpoint, while fear is the dominant emotion in phonophobia, anger is the dominant emotion in misophonia. However, more recent research suggest that other than anger there is at least four other dominant emotions present in misophonia (i.e., irritation, stress and anxiety, aggravation, feeling trapped, and impatience) (Rouw and Erfanian, 2018).

## Incidence

In a recent study, Wu et al. (2014) investigated the incidence, correlates, and impairments associated with misophonia in a student population. Out of 483 undergraduate students (mean age = 21.4 years), 22.8% were often or always sensitive to or annoyed by specific sounds (e.g., eating, repetitive tapping, or nasal noises). Dislike of throat sounds, rustling papers, and environmental sounds were reported by 19.5, 16.1, and 14% of respondents, respectively. Literature suggests that 60% of patients with tinnitus also have misophonia (Jastreboff and Jastreboff, 2002; Jastreboff and Hazell, 2004) and 86% of tinnitus patients have hyperacusis, 25–30% of which requiring treatment (Anari et al., 1999; Jastreboff and Jastreboff, 2006). Jastreboff deduced that 1.75% of the general population has hyperacusis without tinnitus, but it is still difficult to differentiate those who have hyperacusis alone, misophonia alone, and those who have both (Jastreboff, 2015).

## Characteristics

Misophonia usually begins during childhood or adolescence, sometimes affecting academic performance (Edelstein et al., 2013; Schroder et al., 2013). An intense negative emotional reaction is usually triggered by bodily sounds (e.g., chewing, breathing, swallowing, and foot tapping, etc.) and may be connected to a particular person creating that sound (Edelstein et al., 2013; Schroder et al., 2013). In addition to the emotional aversion, patients sometimes report physical pressure building in the chest, the desire to stop the person from making the sound, and other autonomic reactions (Moller, 2011). Sometimes patients will mimic the sound to cancel it out. Rarely do physical reactions, such as assaulting the person making the sound, occur. However, because the patient is never sure when the trigger sound might be heard, the patient often lives in a perpetual state of anxiety. Patients are hyper-focused on listening for that trigger; they will avoid certain situations, people, and foods that they think will cause the sound (Edelstein et al., 2013). Overall, patients may suffer physical and emotional discomfort, contributing to a reduced quality of life (Edelstein et al., 2013).

According to Jastreboff and Jastreboff (2015), only 7 cases (2.2%) out of 318 misophonic patients exhibited a psychiatric disorder. Some researchers argue that misophonia and psychiatric disorders are unrelated. However, others tend to believe that psychiatric disorders and misophonia might coexist. Schroder et al. (2013) conducted a study to classify misophonia as its own form of psychiatric disorder. Their results showed a pattern of intense reactions to specific stimuli, avoidance, and worry that matched with traits of other psychiatric disorders, i.e., social phobia, post-traumatic stress disorder, personality disorders with impulsive aggression, intermittent explosive disorder, autism spectrum disorder, sensory processing disorders, antisocial personality disorder, and phonophobia (Schroder et al., 2013). Although the nosological nature of misophonia is still a topic of debate, Schroder’s findings seem to call for misophonia to be classified as a subtype of a discrete psychiatric disorder.

## Diagnosis

Clinically, diagnosing misophonia requires a detailed case history to determine onset, triggers, reactions, and co-morbid conditions. Questionnaires may also be useful when determining the severity and uniqueness of each patient’s case. Although certain questionnaires have been proposed to evaluate the severity of misophonia (Khalfa et al., 2002; Dauman and Bouscau-Faure, 2005), their validity needs to be confirmed. On the other hand, no one questionnaire has been consistently used across studies for the evaluation of misophonia. Examples of some of the questionnaires currently being used to evaluate misophonia are: (1) the Misophonia Questionnaire (MQ), which is a three-part self-report questionnaire developed by Wu et al. (2014) to assess the presence of misophonia symptoms as well as related emotions and behaviors; and (2) the Amsterdam Misophonia Scale (A-MISO-S), a concept scale based on the already validated Yale-Brown Obsessive-Compulsive Scale (Y-BOCS) (Schroder et al., 2013). The A-MISO-S is a six-item scale that evaluates different areas affected by misophonia such as: the time spent focusing on misophonia; interference with social functions; level of anger; impulse control; control over thoughts and anger; and time spent avoiding situations contributing to misophonia.

The audiological assessment of misophonia is complex. To date there is no agreement on a specified protocol to assess misophonia. However, audiological assessment includes pure tone thresholds and loudness discomfort levels (LDL). Patients with misophonia may have hearing loss or normal hearing. LDLs have been reported at normal and reduced levels (Jastreboff and Jastreboff, 2013). There is no precise description of how to test LDLs in patients with misophonia. It is therefore possible that variations can occur in the results obtained due to the specific method administrated and differences in the way patients are instructed (Hawkins et al., 1987; Sherlock and Formby, 2005; Jastreboff and Jastreboff, 2015). Nevertheless, Jastreboff and Jastreboff (2015) indicated that when misophonia is present with hyperacusis, LDL values can range from 30 to 120 dB HL. This further emphasizes that LDLs alone are insufficient to accurately diagnose hyperacusis and/or misophonia (Jastreboff and Jastreboff, 2015). Differences in auditory late potentials may be present when patients are tested using an oddball paradigm. Schroder et al. (2014) concluded that the deviant tone evoked a smaller N100 in misophonia patients than in healthy controls. Such responses might have been because of deficits in processing auditory information at low intensities or because of coexisting mood and psychiatric conditions. This study supports the recommendation of a thorough case history and use of questionnaires to understand all aspects of the patient’s life that may contribute to misophonia (Ferreira et al., 2013; Schroder et al., 2013).

### Anecdotal Cases in the Literature

A 36-year-old woman, Ms. A, raised in foster homes, had a mother suffering from depression and a father who did not provide support and affection (Veale, 2006). At the time that she visited the clinic, she was married and had one son. Ms. A was a solitary person who desired to be deaf. Her desire was so intense that she would block her ears with cotton balls soaked in oil. Veale (2006) had the patient complete a series of questionnaires and found that she was extremely averse to sounds of any kind and fulfilled criteria for multiple personality disorders. In this case, repeated avoidance of sound might have led to increased auditory gain and worsening of symptoms.

Neal and Cavanna (2013) offered a case study of a 52-year-old man suffering from Tourette syndrome and misophonia. Neuropsychiatric examination revealed multiple motor ticks (e.g., facial grimacing and shoulder shrugging) and phonic ticks (e.g., yelping and barking) since age 11. The man also had mild obsessive-compulsive behaviors, depression, and sleep problems. Interestingly, this man noticed his aversion to sounds (e.g., father chewing food, sounds when riding a bus) developed about 1 year before his tics started. The authors thus speculated that there may be a pathophysiological association between the two conditions.

Webber et al. (2014) reported a pediatric case of misophonia and Tourette syndrome. The young female patient was also diagnosed with comorbid obsessive-compulsive spectrum disorder (OCD) and attention deficit hyperactivity disorder (ADHD). At 6 years of age, she developed frequent motor and vocal tics. During an interview, the young girl reacted strongly to certain auditory and visual stimuli and demanded that the sound stop. These findings are like those reported by Neal and Cavanna (2013), i.e., obsessive compulsive tendencies, an early age of onset, and the presence of both motor and vocal tics. The neural circuitry involved in OCD and Tourette syndrome may be similar to that of misophonia (Husted et al., 2006; Neal and Cavanna, 2013). A methodical screening for misophonia in disorders such as Tourette syndrome and OCD might uncover a pathophysiological connection between sounds and anomalies within the limbic system and its connections with the auditory cortex and the autonomic nervous system.

Kluckow et al. (2014) interviewed 15 patients being treated for eating disorders about possible misophonia symptoms. Three of the 15 patients met the criteria for misophonia. The first patient recalled her misophonia trigger to high-pitched voices starting around age six. Hearing the sound would cause her to binge eat. Coping mechanisms included ear plugs, music for distraction, and digging her fingernail into her hand to cause pain but not draw blood. The second patient had misophonia triggered by the eating habits of family friends. Following the development of her aversion, the patient started to increase exercise and decrease eating. The third patient presented with misophonia caused by the sound of family members eating cereal out of a bowl; she found the clinking spoon in the bowl and the sound of cereal being chewed repulsive. Aversion to these stimuli was so strong that she was unable to eat. In all three cases, severe aversion to eating sounds preceded the development of an eating disorder. Kluckow et al. (2014) suggested that the co-presentation of misophonia and eating disorders should be investigated during a thorough case history.

## Treatment

Currently, there are no research studies that have investigated pharmaceutical options to treat misophonia. Anecdotal information suggests the prescription of antidepressants and anxiolytics to address the reactions and co-morbid conditions associated with misophonia. Despite the lack of pharmaceutical remedies, a variety of therapies have been considered, with some showing signs of potential success.

Tinnitus retraining therapy (TRT) is a therapy program designed by Jastreboff to manage tinnitus and, as a secondary effect, hyperacusis and misophonia (Jastreboff and Jastreboff, 2006). TRT assumes that altering conditioned reflexes at the subconscious level will reduce or eliminate the connection between the auditory system and the limbic and autonomic nervous systems (Kiessling, 1980). Relating these auditory conditions to conditioned reflexes also helps to understand how loudness correlates with severity (Tyler et al., 2007). The connection is reinforced by the two stimuli and the auditory characteristics are of less importance. The therapy protocol consists of directive counseling and sound therapy, tailored to each patient’s specific situation. This model emphasizes the need for the patient to understand the underlying mechanisms of the condition. The patient will adhere to the treatment assigned and make conscious efforts to alter the underlying mechanisms once s/he understands how they operate. A clear review of TRT and individual goals between patient and clinical provider will ensure the patient has realistic expectations. For sound therapy, TRT works to reinforce positive sounds and reduce exposure to sounds causing a negative reaction. Exposure to background noise and avoidance of silence work to desensitize certain sounds. If the patient also has hearing loss, s/he may benefit from ear-level combination devices, which provide amplification and tranquil background sounds. Overall, TRT should take about 9–18 months to complete (Jastreboff and Jastreboff, 2002). If the patient is suffering from hyperacusis and misophonia, Jastreboff and Jastreboff (2015) recommend first treating hyperacusis. Once misophonia is isolated, the goal is to change the relationship between the auditory, limbic, and autonomic nervous systems and to eliminate the conditioned reflex. Although the conditioned-reflex model of TRT has been challenged in theory (Tyler et al., 2006), TRT should work better with misophonia patients than with tinnitus patients because misophonia involves an external trigger, which can be manipulated to potentially eliminate the conditioned response (Jastreboff and Jastreboff, 2002). Misophonia patients undergoing TRT are encouraged to avoid silence and ear overprotection. Decreasing patients’ reactions to their trigger sounds by introducing pleasant sounds and constant low intensity sounds is thought to improve these patients’ conditions. Reclassifying the sounds plus heavy counseling are recommended to help these patients. TRT outcomes were reported by Jastreboff et al., as 152 out of 184 patients with misophonia with hyperacusis, and patients with misophonia without hyperacusis (139 patients out of 167) showed improvements after TRT (Jastreboff and Jastreboff, 2015).

Although the development of a therapeutic approach such as TRT often applied it has not proven to be clinically effective. This has led to some controversy regarding the application of this theory as a therapeutic approach, partially because the underlying mechanism of misophonia is not well understood. Besides TRT, other neuropsychiatric therapies might be effective for misophonia patients. Schneider and Arch (Schneider and Arch, 2015) reviewed potential treatments for misophonia based on specific characteristics of the condition. Since one of the common responses to a misophonia trigger is anger, they suggested focusing on therapies that work to decrease anger, such as cognitive restructuring and stress inoculation training (Blake and Hamrin, 2007). A literature review of therapy and training programs for anger in youth populations indicated that Cognitive-Relaxation Coping Skills Training and Multicomponent Cognitive-Behavioral Therapy (CBT) were able to reduce anger-related behaviors and improve control over the expression of anger (Blake and Hamrin, 2007). Similarly, a stress inoculation training program for high school students significantly decreased negative stress events and reduced overall anger (Hains, 1992). Since misophonic symptoms often arise in adolescence, these anger therapies may also apply to the misophonia population.

Other ways to address misophonia could be through compassion training, distress-tolerance, and acceptance-based treatments (Schneider and Arch, 2015). Mindfulness (moment-to-moment awareness) may enhance traditional CBT programs in psychologically distraught patients (Hofmann et al., 2011). In a group of moderately to severely distressed tinnitus patients, internet-based CBT and acceptance and commitment therapy (ACT) were both shown to reduce the negative impact of tinnitus on quality of life, as measured by tinnitus severity and tinnitus-related distress (Hesser et al., 2014). Techniques used in these programs included applied relaxation, positive imagery, attention training, cognitive restructuring, exposure, and background sounds to cope with tinnitus. There are only two case studies reported in the literature that show the effects of CBT for youths with misophonia (McGuire et al., 2015). After completing psychoeducation, reviewing how to appropriately respond when a trigger is present, and gradually increasing exposure to the trigger sounds, the two female patients had reduced symptoms and expressed that they now had the tools to cope. Although two case studies alone do not constitute concrete evidence that CBT will work with misophonia patients, it does invite further research to determine the efficacy of such a therapy program.

The potential therapies listed above look to address emotional reactions caused by misophonia triggers. In order to have a truly effective treatment, more research is needed to better define a diagnostic protocol, rule out comorbid conditions, and to provide evidence for the underlying mechanisms of misophonia (Webber and Storch, 2015). Since misophonia triggers can be from many sources, people, and situations, it is likely that one therapy program will not address every manifestation of misophonia, indicating a need for individualized therapy.

## Underlying Mechanisms

Auditory information ascends through the brainstem to the cerebral cortices in two parallel pathways, mainly known as the classical and non-classical auditory pathways (Moller and Rollins, 2002). The anatomy of the non-classical pathway differs from that of the classical pathway mainly in the thalamic relay nuclei (Moller and Rollins, 2002). The classical pathway is intermittent in the ventral portion of the medial geniculate body, while the non-classical pathway is interrupted in nuclei located in the medial and dorsomedial geniculate body (Moller and Rollins, 2002; Moller et al., 2005). Recall that misophonia is described as a negative reaction to sound results from enhanced limbic and autonomic responses without abnormal enhancement of the auditory system (Jastreboff, 1999; Jastreboff and Hazell, 1999). Since it is known that classical and non-classical auditory pathways interact with the limbic system, a breakdown in such processes may contribute to an increased association between auditory stimuli and emotional and autonomic reactions (Jastreboff and Hazell, 2004; Langguth and Landgrebe, 2011).

The literature suggests that the majority of patients with misophonia have normal hearing sensitivity (Schroder et al., 2014), while the limbic and autonomic nervous systems are in a heightened state of excitation and thus react abnormally to normal auditory input (Moller, 2011). A recent functional and structural MRI study has revealed that trigger sounds elicited increased responses in the anterior insular cortex (AIC) and abnormal functional connectivity between the AIC and medial frontal, medial parietal, and medial temporal regions (Kumar et al., 2017). The findings of Kumar et al. (2017) implied that there was abnormal myelination in the medial frontal cortex that shows abnormal functional connectivity, and that the aberrant neural response mediates the emotional coloring and physiological arousal that accompany misophonic experiences.

A focal point of rebuttal by the Kumar et al. (2017), study lies in their experimental designs where general annoyance elicited by one stimulus condition (i.e., baby crying, a person screaming) was disassociate from a specifically misophonic reaction elicited by another stimulus condition (i.e., eating and breathing sounds). In a commentary (Schroder et al., 2017), Schroder et al. argued that it was unclear whether the subjects in the Kumar et al. (2017) study actually suffered from misophonia, as Schroder et al. promote the idea that misophonia is a distinct form of a psychiatric disorder with specific and well-defined diagnostic criteria (Schroder et al., 2013). Secondly, the validity of the questionnaire used to select subjects with misophonia were put into question. In addition, anger, which is an essential component in the diagnosis of misophonia, was overlooked and instead the focus was on annoyance. As such, part of Schroder et al.’s argument was that the observed brain differences in the Kumar et al. (2017) study might be correlated to general annoyance rather than anger specifically. A final comment was about the design of the study and how it might have put subjects at risk of sensitization to sound by repeated exposure (Kumar and Griffiths, 2017; Schroder et al., 2017).

In response, Kumar et al. (2017) stated that, to date, there are no diagnostic criteria for misophonia in ICD 10 or DSM-5, as subjects were selected based on having stable typical response to trigger sounds over years which are usually anger but can also come in the form of anxiety. This argument was supported by findings of a large scale study involving more than 300 subjects with misophonia, who primary reported emotional responses in the form of irritation/annoyance and not anger (Kumar and Griffiths, 2017). Finally, regarding Schroder et al.’s last comment, Kumar argued that it was not clear how re-exposure to sounds that have been producing a typical misophonic reaction for years might have any bearing on the reaction produced (Kumar and Griffiths, 2017). Despite the controversy surrounding these findings, the AIC is one of the core components of limbic and autonomic nervous system activity control.

Learning involves associating events happening at different times, a process that is of fundamental importance for a number of perceptual and cognitive processes (Wallenstein et al., 1998; Fuster et al., 2000). There are two forms of learning, associative and non-associative, which we will briefly describe and then use to elucidate misophonia. Associative learning involves one stimulus presented simultaneously with another stimulus, creating a specific reaction. Conditioning to stimuli can be either through classical or operant conditioning (Vlaeyen, 2015). Non-associative learning is a change in behavior after repeated presentations of a stimulus, but there is no reinforcement via a second stimulus like there is in associative learning. In response to a single stimulus, an individual can either experience habituation or sensitization. Habituation is a decrease in response to a stimulus following multiple identical presentations (Ursin, 2014). In healthy systems, habituation and sensitization counteract one another and allow the individual to stay in a neutral state (Ursin, 2014). Associative learning, particularly classical conditioning, and non-associative learning, particularly sensitization, may help to explain the underlying mechanisms of misophonia.

### Associative and Non-associative Learning

Jastreboff and Jastreboff (2002) developed a model to explain the neural mechanisms governing tinnitus, hyperacusis, and misophonia (Jastreboff and Jastreboff, 2002). Associative learning via classical conditioning supports their theory. Since classical conditioning works in anticipation of a change to the environment (Vlaeyen, 2015), they hypothesized that patients suffering from one or all three of these problems have enhanced connections between their auditory system and their limbic and autonomic nervous systems (Jastreboff and Hazell, 2004). Misophonia is a form of conditioned behavior that develops as a physical reflex through classical conditioning with a misophonia trigger (e.g., eating noises, lip-smacking, pen clicking, tapping and typing …) as the conditioned stimulus, and anger, irritation or stress the unconditioned stimulus. The involvement of the limbic system helps to explain the emotional component to this condition and suggests that the connection is controlled by a conditioned reflex. Three components of the limbic system (the amygdala, parahippocampus, and insula) have been shown to activate more strongly in other conditions such as tinnitus (Song et al., 2013, 2015; Carpenter-Thompson et al., 2014). Moller (2013) also suggested that the amygdala’s control of fear, depression, and anxiety may explain a connection to the auditory system in patients with phonophobia.

Activating the limbic and autonomic nervous systems triggers irrational reactions to stimuli (Molini et al., 2014). Hyperacusis patients have increased auditory sensitivity, which is passed on to the autonomic nervous system and results in increased activation (Jastreboff and Jastreboff, 2015). Similarly, misophonia patients have an increase in activation between the auditory pathways and autonomic nervous system, resulting in negative emotional reaction to sound. Similarly, pain processing involves an association with intrusion, long-term exposure, depression, anxiety, defensive responses, and prolonged avoidance (Vlaeyen, 2015). Misophonia patients have triggers that cause annoyance, anxiety, and depression. They respond by trying to ignore or escape the stimulus. Prolonged avoidance can exacerbate the condition. Misophonia patients may plug their ears with cotton balls or live a life of silence (Veale, 2006; Edelstein et al., 2013). As in pain, the trigger is not life-threatening, and yet it leads to worry, fear, and anxiety. Although these points should not be treated as specific to misophonia, further investigation into tinnitus, hyperacusis, and pain mechanisms may also help to understand misophonia.

This begs the question of why some people learn this type of association (classical conditioning) and not others. Classical conditioning or associative learning elicits reflexive, automatic, and involuntary behavior (Jarius and Wildemann, 2015; Kotchoubey and Pavlov, 2017). The only responses that can be elicited out of classical conditioning are those that rely on responses that are naturally made by the individual with misophonia or, for that matter, any other condition. These responses are often involuntary and occurring below the level of conscious awareness (Jarius and Wildemann, 2015). The hallmark of misophonia is an extreme emotional response to the trigger stimulus. As a result, for misophonic patients, these emotional responses might create a classical conditioning paradigm that maintains or strengthens the misophonic physical reflex (Schroder et al., 2013). With that said, individual differences in associative learning do exist, in part due to psychological and individual personality variables (Murphy and Msetfi, 2014).

On the other hand, non-associative learning can result in habituation or sensitization. The symptoms of misophonia arise from enhanced sensitized functional connections or shortcuts between the limbic, auditory, and autonomic nervous system (Schwartz et al., 2011). Sensitization is defined as increased neuronal activity in response to a stimulus (Jimenez et al., 2017). Before stimuli or neural activity reach the brain, they are classified and evaluated by complex neuronal pathways. If the signal becomes actively connected to previous emotions or memories, there will be an overlap in the auditory signal and emotions, creating a complex neural signal. That complex neural signal is what finally reaches the level of conscious awareness; hence, the anxiety and stress related to the auditory signal are incorporated subconsciously and revealed at the conscious level. After repeated cycles of this process, the neuronal reaction threshold decreases and allows the complex hyper-responses to reach the brain more easily (Zenner et al., 2006). The underling mechanism for sensitization in misophonia is unknown, but has typical been associated to strengthening of synaptic signals, a process known as long-term potentiation, or “kindling,” repeated stimulation of specific neurons in the limbic system. In a recent publication showed that misophonia patients can have enhanced autonomic reactivity to a sound, but not to other sensory stimuli (Edelstein et al., 2013). The subjective experiences described by these misophonia patients to trigger sounds share qualitative features with the sensory symptoms reported by patients with tic disorders such as Tourette syndrome. Recent reports have also suggested that misophonic symptoms can be found in the context of two of the most common psychiatric comorbidities of Tourette syndrome, in addition to obsessive-compulsive disorder, generalized anxiety disorder, and schizotypal personality disorder (Ferreira et al., 2013; Neal and Cavanna, 2013; Cavanna and Martino, 2014). Overall, although there is preliminary evidence supporting the suggestion that the underlying mechanism can occur in misophonia due to sensitization more research is needed.

### Association Between Misophonia and Synesthesia

The brain constantly integrates signals across different modalities. To that extent, a defining aspect of misophonia occurs when the disturbing sound produced by others provoke an emotional response. This emotional response exhibits a marked connection between the auditory system and other limbic and autonomic systems (Bruxner, 2016). This process resembles another phenomenon: synesthesia which is the occurrence of a particular sensory stimulus can evoke additional sensations and associations (Galton, 1883; Harrison and Baron-Cohen, 1995; Baron-Cohen et al., 1996; Barnett et al., 2008; Edelstein et al., 2013). It is proposed that synesthesia results from an increase of neural connections and interactions between different sensory modalities (Brang and Ramachandran, 2011; Mylopoulos and Ro, 2013).

There may be a connection between misophonia and synesthesia. In synesthesia, as in misophonia, a pathological distortion of connections between the auditory cortex and limbic structures can cause a form of sound-emotion synesthesia (Edelstein et al., 2013). Furthermore, in both phenomena, an external sound can produce internal perceptual and sensational experiences (Barratt and Davis, 2015). There are also reports suggesting that the two phenomena are linked by their affective components, in addition to their perceptual similarities (Edelstein et al., 2013). For example, negative autonomic reactions are associated with the experience of misophonia. Specifically, sufferers report that noises of any volume made by others such as breathing, swallowing, or foot tapping can elicit feelings of disgust, anger, or hatred (Schroder et al., 2013). Misophonic experiences are also similar to synesthetic associations in that they are both automatic and cross-modal. Further exploration of the similarities between these two conditions is needed to discover whether and how these two phenomena are related.

## Conclusion

Certain characteristics of misophonia that follow rules from both associative and non-associative learning principles could possibly be used to better understand the underlying mechanisms. If non-associative learning does help to explain the underlying mechanisms of misophonia, then there needs to be research investigating this connection. To date, research has made only weak speculation, with little evidence to support the theory. TRT seems to be an effective treatment option for patients with sound sensitivity disorders such as misophonia. Although the majority of patients do find relief through TRT, there are still cases that receive no relief. Finally, Vlaeyen (2015) suggested a connection between non-associative and associative learning. The anxiety evoked by a stimulus may induce negative effects, causing sensitization. Perhaps this is the key to a successful misophonia treatment. By combining TRT and sensitization strategies, those few patients who do not receive relief via either method alone might benefit from a combined method. Future studies should focus on further examining the relationship between associative and non-associative learning and misophonia.

## Author Contributions

All authors listed have made a substantial, direct and intellectual contribution to the work, and approved it for publication.

## Conflict of Interest Statement

The authors declare that the research was conducted in the absence of any commercial or financial relationships that could be construed as a potential conflict of interest.
